# Conditionally Reprogrammed Cells and Robotic High-Throughput Screening for Precision Cancer Therapy

**DOI:** 10.3389/fonc.2021.761986

**Published:** 2021-10-19

**Authors:** Faris Alkhilaiwi

**Affiliations:** ^1^Department of Natural Products and Alternative Medicine, Faculty of Pharmacy, King Abdulaziz University, Jeddah, Saudi Arabia; ^2^Regenerative Medicine Unit, King Fahd Medical Research Center, King Abdulaziz University, Jeddah, Saudi Arabia

**Keywords:** conditional reprogramming, robotic HTS, *in vitro* assay, cancer, precision medicine

## Abstract

Cancer is a devastating disease that takes the lives of millions of people globally every year. Precision cancer therapy is based on a patient’s tumor histopathology, expression analyses, and/or tumor RNA or DNA analysis. Only 2%–20% of patients with solid tumors benefit from genomics-based precision oncology. Therefore, functional diagnostics and patient-derived cancer models are needed for precision cancer therapy. In this review, we will summarize the potential use of conditional cell reprogramming (CR) and robotic high-throughput screening in precision cancer medicine. Briefly, the CR method includes the co-culturing of irradiated Swiss-3T3-J2 mouse fibroblast cells alongside digested primary non-pathogenic or pathogenic cells with the existence of Rho-associated serine–threonine protein kinase inhibitor called Y-27632, creating an exterior culture environment, allowing the cells to have the ability to gain partial properties of stem cells. On the other hand, quantitative high-throughput screening (qHTS) assays screen thousands of compounds that use cells in a short period of time. The combination of both technologies has the potential to become a driving force for precision cancer therapy.

## Introduction

Cancer is considered the first or second cause of death in more than 100 countries around the world in 2019 for people younger than 70 years (1). There are more than 10 million cancer deaths and more than 19 million newly diagnosed cancer cases in 2020 (2). Clinical guidelines for treating different diseases such as hypertension, diabetes, and cancer have been established by panels of experts in health organizations such as the European Society for Medical Oncology (ESMO) and the American Society of Clinical Oncology (ASCO) (3). The reasons why experts make these guidelines are the large number and confusion in some of the medical studies toward what is the best treatment; some cancers are rare, which makes their treatment by inexperienced physicians difficult and makes bias by physicians unavoidable, which is intrinsic in medical practice. These guidelines for cancer management should be backed up with clinical trials. However, these are often lacking, and therefore some cancer guidelines represent only a “pattern of practice,” often that of large medical institutions with a large expertise and experience in those cancers (3).

However, it has become increasingly clear over the past decade that no two patients’ cancers are exactly the same and, therefore, may have different responses to generic treatments such as radiation and chemotherapy (4). This classical model for cancer therapy is overly simplified; it results in expensive treatments and ineffectiveness and causes patients to suffer from unnecessary side effects (5).

## Genomic Medicine for Precision Medicine

The basic strategy in genomic medicine is to identify somatic genetic changes such as quantitative chromosomal abnormalities, amplifications, translocations, and point mutation and match it with medications targeting these defects for a patient’s advantages. Within certain conditions, a large number of patients who have specific types of tumors carrying a specific mutation have been treated with a single medication successfully (6). The utilization of imatinib to treat patients with chronic myeloid leukemia (CML) carrying the t(9;22) translocation that produces the BCR-ABL fusion kinase was the first example (7). Another example is using epidermal growth factor receptor (EGFR) inhibitors successfully to treat lung cancers that harbor mutant EGFR, also using BRAF inhibitors to treat the melanomas carrying mutated BRAF (8–11). Recently, a significant chance for an immune checkpoint inhibitor was specified in Hodgkin’s lymphoma by genetic mode (12, 13). For every patient who has lung cancer sheltering a targetable mutation in ALk or EGFR, there are four who lack any targetable mutation (14).

Although clinical trials did not evaluate the utilities, it still has gained us an assessment tool for patients to target therapies through genomic techniques. In May 2016, a release of interim analysis from the molecular analysis for therapy choice (NCI-MATCH) stated that there was good analytical achievement in their genetic screening technique, while testing was done in 87% of the 739 samples. Nonetheless, 9% of the patients of whom their testing was accomplished had tumors that harbored a mutation that may guide them to any of the 10 targeted therapy arms, and a slight percentage around 2.5% has managed to get in a treatment arm (6). The University of Texas MD Anderson Cancer Center conducted a study where 2,000 tumors from 2,000 patients were investigated with platforms containing 11, 46, or 50 genes. From these tumors, 789 (39%) had possibly targetable alterations; however, only 83 (4.2%) of the patients were included in a genotype-matched trial (15). Unfortunately, the clinical effect was not reported. A recent report by the Dana-Farber Cancer Institute where they use massively parallel sequencing assay including 282 genes for 3,727 patient tumors stated that 73% of these tumors contain an “actionable or informative alteration” (16). There are individual cases where clinical benefits appear to be clear, but still no full evaluation of the clinical benefit is reported. Marquart et al. evaluated 31 drugs with 38 Food and Drug Administration (FDA)-approved indications between 2006 and 2018. In 2006, the percentage of metastatic cancer patients who were evaluated and found to have a positive clinical outcome from the genome-targeted therapy was 0.70%. However, the percentage had risen to 4.90% in 2018 (17). The author concluded that genome-driven drugs had benefited only a small number of patients with advanced cancer (fewer than 7%) even though an increased number of patients became eligible (yet fewer than 16%) (17).

## *In Vitro* Drug Sensitivity Assay for Precision Medicine

During the course of treating cancer, initially, cancer cells may respond to medications; however, after a period of time, tumor cells change to become resistant as a result of mutation of cellular genes (18). The genetic and molecular profiles of the pathological tissues such as the cancer tissues need to be adopted and analyzed prior to the interference with the new medications. Nevertheless, in some rare cases, there are unsolved genetic alterations that may be hard for medication to target (18). Ideally, prior to the start of certain treatment, it is propitious to carry out an *in vitro* drug sensitivity assay to investigate whether they show an inhibitory effect on the primary cancer cells from the cancer lesion itself (19).

With the acknowledgement of the limitations of existing cancer models, numerous patient-derived models of cancer (PDMCs) are in development. Recently, PDMCs like induced pluripotent stem cells (iPSCs), conditionally reprogrammed cell cultures (CR), patient-derived xenografts (PDXs), organoids, spheroids, and others have been established (**Table 1**) (20). And in any other prototypes or model system, every single platform has its own pros and cons when it comes to their usefulness and also their illustration of tumor structure, microenvironment, stem-differentiation states, cellular conformation, growth patterns, heterogeneity, and responses to therapeutics; all of these advantages and disadvantages are mostly dependent on the samples and specimens drawn from the patient (20). These approaches are under assessment for further use in the future in hope to understand the basic biology that lays behind cancer and the benefits in translational cancer research, specifically for target identification, drug screening, and the discovery of biomarkers that are key for detecting cancer at early stages (5).

**Table 1:** Comparison of patient-derived cancer models CR, organoids, iPSCs, and PDX.

	Conditional reprogramming	Organoids	Tumor-derived iPSCs	PDX
Success rate of initiation	High	Moderate–high	Moderate	Moderate
Derivation time	Days	Weeks	Weeks	Months
Expansion	Very fast	Moderate–fast	Moderate–fast	slow
Ease of maintenance	High	Moderate	Moderate	Low
Representation of heterogeneity	Moderate	Moderate	Moderate	High
High-throughput screens capability	High	Moderate	High	Not applicable
Microenvironment	Possible with co-culture	High	Possible with co-culture	Very high
Representation of tumor	High	High	High	Very high
Cost	Low–medium	High	High	Very high

iPS, induced pluripotent stem; PDX, patient-derived xenograft.

### Induced Pluripotent Stem Cells

iPSCs can be used to identify a link between drug responses and genotype and to recognize biomarkers to guide patient selection for clinical trial enrollment (21, 22). For instance, Cao et al. established iPSC-derived sensory neurons from patients with inherited erythromelalgia (IEM); the reversal of hyperexcitability in these cells using a selective sodium-channel blocker did correlate with the specific mutations and the clinical response in those patients (22).

### Circulating Tumor Cells

Over the past few years, protocols for isolation and downstream application of circulating tumor cells (CTCs) from cancer patient’s blood have significantly improved. Preceding methods needed immediate grafting of CTCs into immunocompromised mice for propagation or had low success rates (23–25). Lately, CTC microfluidic chips were used to isolate CTCs from breast cancer patients; these chips were shown to enrich tumor cells for additional manipulation (26). Supporting the growth of these isolated cells needs unique culture conditions; interestingly, growth of CTC in culture was only achievable when the cells were grown as a suspension, not as adherent cultures, which is in agreement with their circulating phenotype. Drug screening showed that the CTC cells were consistent with the patient’s clinical response when they maintained functional responses to chemotherapies and moreover proposed novel drug combinations (26). Given the simple access to patient blood for isolation of CTCs and their use for drug screening, they represent a captivating source for patient tumor cells for ultimate downstream functional testing. However, propagation of CTCs from the typically <100 cells isolated from patients’ blood do require months (sometimes no cells can be isolated) to secure enough cells for any type of testing such as drug screening, during which the primary tumor in the patient may no longer be represented by the propagated CTC population (27).

### Organoid Cultures

The labs of Kuo, Cleversand, and others were the first to use mouse tissue to establish organoid cultures (28–33). These results have been widened to tumor and normal tissues from human samples as well. Organoid cultures come up with principles to explore and understand the biology of premature phase cancer to investigate drug resistance and identify drug targets (32, 34–41). Contrary to the monolayer cell lines that are developed in the flask on plastic, organoid cultures are established by growing epithelial cells in matrigel in a three-dimensional manner. These cultures have many advantages; for instance, they capture the original tumor heterogeneity, are genetically stable, and can be grown for many passages and for a long time, which allow us to conduct many experiments. Both normal and cancer organoids can be achieved from the exact patient samples, which provides an opportunity for precision therapy. Since the organoid culture system grows both cancer and normal cells, error-free tumor tissue sampling is highly important and critical. Moreover, these cultures are more convenient for low-throughput drug screening rather than the high-throughput one (40, 41). Generally, organoids consume 4–6 weeks to get enough cells for the screening (42).

### Patient-Derived Xenografts

In the past few years, PDXs have arisen as a beneficial system for *in vitro* modeling of human tumors (43, 44). Dissimilar to cell lines that have many disadvantages such as absence of stromal components, genetic drift because of the extended period of culturing condition, and clonal selection, the PDX model preserves the cross-talk between the epithelial tumor cells and the stromal components, maintain the cellular and molecular heterogeneity of the primary tumor, and have high genetic stability mainly in the first few passages. Additionally, PDX model seems to anticipate the potential of metastasis to occur and the response to treatment (45). At this time, PDXs are highly identified as more relevant preclinical models from a physiologically point of view compared with classic cell lines (45). PDX models successfully recapitulate the tissue histology and the gene expression profile of the original tumor. In contempt of their benefit, the absence of growth *in vitro* and lower throughput limit and narrow the use of PDX models. Nevertheless, *ex vivo* genetic manipulation, high-throughput chemosensitivity screens, and the development of novel orthotropic models can be accomplished by developing cell lines from the PDXs. However, establishing cell lines from PDXs is still an obstacle because of the outgrowth of murine stromal fibroblasts, narrowed differentiation capacity, and lineage commitment. Comprehensively, it takes 2–5 months at proportionately high cost for PDX expansion (46).

### Conditionally Reprogrammed Cells

**Figure 1:**
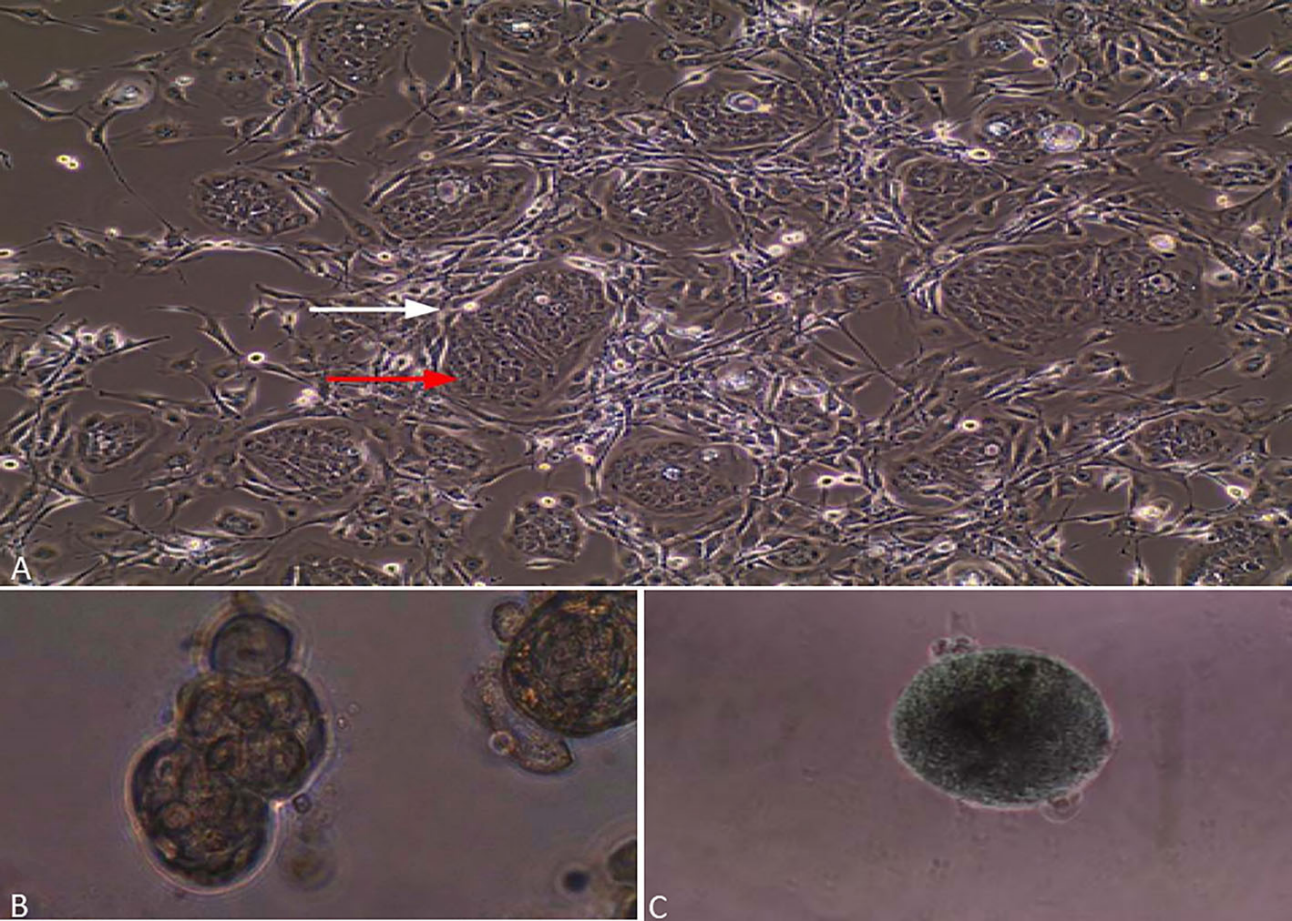
Lung cancer cells grown by CR method. **(A)** Red arrow is assigned to epithelial cancer cells, while a white arrow is assigned to irradiated fibroblasts. **(B)** Lung cancer cells grown in matrigel and forming spheres. **(C)** Lung cancer cells grown in ultra-low-attachment dishes and forming 3D multicellular tumor spheroids (MCTS).

Until the CR method development, it was difficult to make a fast and easy-to-perform method that has a high success rate in a single model system. The CR method that was developed at Georgetown University satisfies the above criteria by being fast and easy and having a high success rate (47). Basically, the culture process does change the condition of normal and tumor cells to a condition of “reprogrammed stem-like,” which makes cells highly proliferative and maintain the original karyotypes of the cells. Also, when the conditions that reprogrammed the cells are taken out, these cells restore the capacity to differentiate (47–49). Hence, the name “conditional reprogramming” was given to the method. CR method is a process that starts by taking a biopsy sample from the patient and then evaluated by the pathologist histologically to define the biopsy components (i.e., define the percentage of cells that are tumor cells). Then we digest the biopsy enzymatically and mechanically, plate it in a medium consisting of irradiated Swiss-3T3-2J mouse fibroblasts (feeder cells) and 10 μM in Y-27632, and use the media with the sample after dispersing into a single cell (**Figure 1**) (47). CR method is available to many epithelial tissues including skin, prostate, lung, and many tissues. Also, its use is not limited to humans; it can be used on many mammals such as rats, dogs, cows, mice, and horses (50–54). One of the most important features of CR cultures that can be used for generating xenograft (47, 48) and PDX cell lines (55) also may be used to establish cell cultures from PDX and organic cultures. Lastly, CR cells keep cell lineage commitment and retain heterogeneity of cells present in a biopsy (47, 48, 56–59).

The first use of CR for precision medicine was by Yuan et al. when they reported a case of a patient with progressive recurrent respiratory papillomatosis (RRP), a condition caused by human papillomavirus (HPV), which is resistant to chemotherapy. Yuan acquired both normal and tumor tissues from the patient and used conditional reprogramming technology to create paired cell lines to be used for identifying possible novel treatment strategies (60). As an outcome, vorinostat revealed a significant and differential cytotoxic effect on conditional reprogrammed tumor cells in comparison with normal cells. The patient started a 3-month course of therapy with vorinostat that led to a stable disease. That was the first “proof of concept” that conditional reprogramming excellently assists in fast propagation of the cancer cells without changing their genetic profile, which makes it a precious tool for *in vitro* sensitivity assays that help physicians to choose the right medication for each patient (60).

Conditional reprogramming culture of various other main types of cancer such as salivary gland cancer, breast cancer, bladder cancer, and lung cancer have also been established and utilized for examination of drug sensitivity (47). Li et al. generated and characterized 14 conditional reprogrammed lung cancer cells, with six cases tested for vinorelbine, cisplatin, carboplatin, and nedaplatin exhibiting congruity with clinical scenario (61). Comparably, other reports showed that conditionally reprogrammed cells from the primary tumor allowed for fast screening of candidate medications and promoted precision therapy (**Table 2**) (50, 56, 57, 64, 65). Additionally, in a recent research, Mimoto et al. worked on luminal-B breast cancer, which has poor prognosis due to lack of appropriate targeted treatment. The authors used the primary tumor as sources for CR cells to establish a xenograft model where both the cells and the xenograft were used for drug sensitivity evaluation (66). Furthermore, conditional reprogramming helped to recognize and explore new treatment strategies. By using patient-derived conditionally reprogrammed prostate cancer cells, it was discovered that LA-12 improves cell death caused by TRAIL, a member of tumor necrosis factor (TNF) family 168, and combinatorial therapy of TRAIL with LA-12/cisplatin killed prostate cancer cells more successfully (67), which provides implications for new combinations of medications for prostate cancer. In addition, Crystal et al. established cells using CR technology from a non-small cell lung cancer (NSCLC) that are resistant to tyrosine kinase inhibitors; such cells allowed the screen of totally new active and novel drug combinations. For instance, in an EGFR mutation-driven resistant cancer that was exhibiting a novel mutant, FGFR combined suppression of FGFR and EGFR was effective in inhibiting cancer growth (68). To investigate the mechanism of nab-paclitaxel-resistant in pancreatic ductal adenocarcinoma (PDAC), Parasido et al. used CR technology to generate nab-paclitaxel-resistant (n-PTX-R) cells and then determined that sustained induction of c-MYC in the n-PTX-R cells is driving nab-paclitaxel resistance in PDAC (69).

**Table 2:** Applications of conditional reprogramming in precision medicine.

Tissue	Tumor types	Therapy type	Finding	Reference
Airway (laryngeal)	Recurrent respiratory papillomatosis (RRP)	Chemotherapy	*In vitro* culture identifies vorinostat as a treatment and showed positive clinical response	(60)
Lung or pleural effusions	Non-small cell lung cancer (NSCLC)	Targeted therapy	*In vitro* sensitivities reflect the clinical response	(62)
Colon	Colorectal cancer (CRC)	Chemotherapy	*In vitro* sensitivities reflect the clinical response	(63)

Taken together, conditional reprogramming offers new advantages in clinical precision therapy, especially for some cases that showed unsolved genetic profile, medication resistance, and no successful therapeutic options.

## Quantitative High-Throughput Screening

Since the emergence of high-throughput screening (HTS) in drug discovery, it went through fast strides and established itself as one of the many premises of the present research in biology and chemistry (70). In 2006, Inglese et al. came up with a quantitative HTS (qHTS) strategy in order to enhance screening precision and accuracy; in a manner, the assays are conducted with numerous compounds’ concentrations in a magnitude ranging at more than four orders to create dose–response curves, which are in all of the tested compounds (71). This method of qHTS has led to an increase in the accuracy of screening compared with the old traditional HTS, since there is no longer a need for the primary single-concentration screening step that was known for its possibility of producing a high rate of false negatives and false positives (72).

During the past 10 years, the HTS technique has been advancing thanks to improvements in many technologies such as the establishment of innovative platforms, automation of liquid handling, and the improvement of tools that are capable of analyzing big sets of data. Hence, the HTS has become critically essential for all phases in the drug discovery journey from bench to bedside, starting from the target detection to the toxicity assessment. Miniaturization and automation help to decrease the use of reagents and the time required for analysis and also to reduce the need for labor-intensive steps, therefore resulting in decreasing the total assay cost by a large margin and making it economically and timely suitable.

The qHTS screening using numerous concentrations of thousands of different compounds constitutes a spectacular progress of success by decreasing the false-negative outcome when compared with single concentrations in the classical HTS (71). In drug discovery and toxicological studies, the generated data from qHTS have a leading role in advancing these two fields (73–75). For example, the concentration or dose-dependent response data are now being created and published for hundreds of toxicologically relevant endpoints in the second phase of the tox21 alliance between the FDA, the Environmental Protection Agency (EPA), National Center for Advancing Translational Sciences (NCATS), and the National Toxicology Program (NTP) (76). The results obtained from these qHTS tests could be applied for many other different purposes, such as screening of phenotypes, prediction modeling, and genome-wide association mapping (77–79).

## CR Technology and Robotic Quantitative High-Throughput Screening

Alkhilaiwi et al. conducted the first proof-of-concept study to merge the CR technology and robotic qHTS. In the study, we were able to propagate millions of cells from a lung tumor biopsy in 21 days and send the cells to the NCATS where they screened the cells using two libraries. The rapid expansion of 35 million cells of the RRP CR cells met the demand for a large number of testing cells.

**Figure 2:**
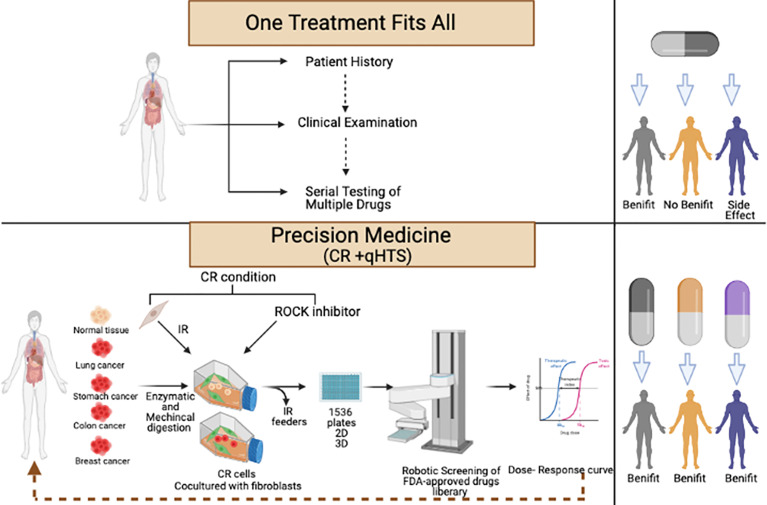
Workflow of conditional reprogramming (CR) technology and robotic quantitative high-throughput screening (qHTS) for precision medicine.

Testing of 4,700 drugs with seven to 11 concentrations each was performed in in a week to identify the potential treatments for the patients who were alive during the study. Three medications have been identified as effective and were validated in 2D culture and 3D sphere cultures. Lowering the number of the medications tested using specific libraries based on the type of the cancer will shorten the time needed to get the result since less cells are needed (**Figure 2**) (80).

## Discussion

In spite of the clear fact that patient physiology differs considerably from one person to another, patient care and drug development depend mainly on the administration of the exact treatment options to diverse populations (19). This created a huge unmet need leading to an act that was signed into law in the United States at the end of 2016 (The 21st Century Cures Act). The act provides funding and resource to the FDA to establish new initiatives and programs that will increase its ability for fast approval of precision medicine products such as medical devices (breakthrough devices) and cell therapies.

Although there are very important features and uses of CR technology in different areas, CR still has many challenges, and the optimization of CR culture is needed when growing different types of primary cells. The first challenge with CR is the inability of maintaining a culture of certain malignant primary cells. In 2017, Yu et al. hypothesized that CR preferentially supported the outgrowth of non-malignant epithelial cells from nasopharyngeal carcinoma biopsy (81). Another group has observed similar results on primary culture of NSCLC tumor specimens (82, 83). In the study of Hynds et al., CR culture was able to establish tumor cell culture from one out of 10 primary NSCLC tumors. They advised that outgrowth of cancer epithelial cells by normal epithelial ones in late passages and cancer cells was seen at early passage only (passage 2) (84). Remarkably, these CR tumor cultures were able to form xenograft when injected in immunodeficient laboratory mice (NSG mice), and the cells that were re-cultured from tumor xenograft were found to maintain the main features of primary tumor (84). Nonetheless, many studies that have been published have described culturing tumor cells from NSCLC (68, 85). On the other hand, there were no reports for outgrowth of normal cells in neuroendocrine cervical cancer and pancreatic cancer (69, 86). The divergence in the results from the different studies could be contributed by different origins of fibroblasts, which were used as feeders in the CR culture, different methods of processing, and acquiring the primary tissues and errors in the pathology laboratory assessment.

The second challenge with the CR method is the inability to discriminate tumor cells from normal populations, which is problematic since some tumor tissues are mixed with normal tissues (47, 85). Such a problem manifests when normal cells grow with the cancer cells from the tumor/normal tissue and occasionally normal cells outgrow the cancer cells as detailed previously (47). To solve this problem, the pathologist needs to assess and designate carefully the normal and tumor tissues (47). Optimizing culture conditions can be performed to favor the growth of prostate tumor cells by removing serum from the medium, which leads to differentiating the normal cells, while prostate tumor cells grow as a mesenchymal morphologic phenotype and then reversed back to epithelial morphology in CR condition (51). Lou’s group suggested to use 3D culture and CR to reach the best results for the differentiation of normal cells to occur and therefore allowing for discrimination between normal and tumor cells (87). Another modification in the culturing condition could be the use of human fibroblasts as feeder cells rather than mouse fibroblasts since the human fibroblasts do not support the *in vitro* proliferation of normal epithelial cells in the long term, which will lead to selecting tumor cells to grow (62, 68, 88). Technologies such as tumor-specific antibody and next-generation DNA sequencing can be performed to differentiate between normal and cancer cells as well. One of the main advantages of CR culture is the ability to form 3D culture, allowing for imitating and recapitulating the *in vivo* condition and the ability to add more cells from the microenvironment of cancer such as immune cells or fibroblasts. Transwell dish-based 3D models from prostate CR cancer cells, organoid culture of prostate CR cancer cells, and multicellular spheroids of laryngeal, lung, and salivary cancer CR cells have been generated (50, 51, 80, 89). Additionally, the CR technique can be used for direct isolation of CTCs from the blood (90, 91).

Naeem et al. have used computational proteochemometric platform (DrugGenEx-Net) to identify carfilzomib for prostate cancer based on transcriptomic data from two matched pairs of benign and tumor-derived CR cells; such system can be valuable in building the library for each cancer in case the number of medications tested needs to be decreased to save more time (92). Moreover, the generated data from each screening will be fed back into the system to help enhance the addition or removal of drugs from the libraries of each disease. In conclusion, there is a huge potential for combining CR and robotic qHTS toward precision medicine based on the patient cells and therefore satisfy a huge unmet medical need.

## Author Contributions

Conception and design, development of methodology, acquisition of data, writing, review, and/or revision of the manuscript: FA.

## Funding

This project was funded by the Deanship of Scientific Research (DSR) at King Abdulaziz University, Jeddah, under grant no. (G-712-166-1441). The author, therefore, acknowledges with thanks DSR for the technical and financial support.

## Conflict of Interest

The author declares that the research was conducted in the absence of any commercial or financial relationships that could be construed as a potential conflict of interest.

## Publisher’s Note

All claims expressed in this article are solely those of the authors and do not necessarily represent those of their affiliated organizations, or those of the publisher, the editors and the reviewers. Any product that may be evaluated in this article, or claim that may be made by its manufacturer, is not guaranteed or endorsed by the publisher.

## Abbreviations

CR, conditional reprogramming; qHTS, quantitative high-throughput screening; NSCLC, non-small cell lung cancer; NSG mice, NOD scid gamma mouse; NCATS, National Center for Advancing Translational Sciences.
